# The Impact of Food Specific IgG Antibodies on Migraine and Its Comorbidities

**DOI:** 10.1002/iid3.70056

**Published:** 2024-11-17

**Authors:** Zhi‐Ming Zhao, Mei‐Mei Yang, Xian‐Shu Zhao, Fu‐Jun Wan, Bao‐Li Ning, Li‐Ming Zhang, Jun Fu

**Affiliations:** ^1^ Health Center of Screening and Prevention of Diseases First Affiliated Hospital of Harbin Medical University Harbin Heilongjiang China; ^2^ Department of Neurology Fourth Affiliated Hospital of Harbin Medical University Harbin Heilongjiang China; ^3^ Department of Gastroenterology First Affiliated Hospital of Harbin Medical University Harbin Heilongjiang China; ^4^ Department of Neurology First Affiliated Hospital of Harbin Medical University Harbin Heilongjiang China

**Keywords:** anxiety, depression, food specific IgG antibodies, gastrointestinal symptoms, migraine, sleep disorders

## Abstract

**Objective:**

To investigate the differences in headache and comorbidity symptoms between migraine patients with negative and positive food specific IgG antibodies, and explore the correlation between these symptoms with food specific IgG antibodies.

**Methods:**

A total of 129 migraine patients were enrolled. Seven questionnaires were used to gather information regarding the symptoms of migraine, gastrointestinal, depression, anxiety, and sleep. Serum specific IgG antibodies against 14 kinds of food were detected using enzyme‐linked immunosorbent assays.

**Results:**

Patients with migraine diagnosis who had positive food specific IgG antibodies had significantly worse headaches, gastrointestinal and anxiety symptoms, compared to the patients with negative IgG antibodies. Patients with more IgG positive foods and higher total positive IgG concentration generally had worse migraine conditions, anxiety, depression, and gastrointestinal symptoms.

**Conclusion:**

The effect of food specific IgG antibodies on severity of migraine and its comorbidities were antibody‐quantity and IgG‐concentration dependent. Future studies are warranted to explore the mechanism underlying such relationship.

## Graphical abstract

Patients with migraine diagnosis who had positive food specific IgG antibodies had significantly worse headaches, gastrointestinal and anxiety symptoms, compared to the patients with negative IgG antibodies. Patients with more IgG positive foods and higher total positive IgG concentration generally had worse migraine conditions, anxiety, depression, and gastrointestinal symptoms. The effect of food specific IgG antibodies on severity of migraine and its comorbidities were antibody‐quantity and IgG‐concentration dependent.

**Abbreviations**
BMI: Body mass index
COVID‐19: Corona Virus Disease 2019
ELISA: enzyme linked immunosorbant assay
GSRS: Gastrointestinal Symptom Rating Scale
HIT‐6: Headache Impact Test‐6
IgG: Immunoglobulin G
MIDAS: Migraine Disability Assessment questionnaire
MSQ: Migraine‐Specific Quality of Life Questionnaire
PSQI: Pittsburgh Sleep Quality Index
SAS: Self‐Rating Anxiety Scale
SDS: Self‐Rating Depression Scale
VAS: Visual Analog Scale

## 1 Introduction

Migraine is the second most common neurological disorder in the world. Its incidence shows an overall increasing trend in China [1]. The estimated 1‐year prevalence of migraine in China was 9.3%, not dissimilar from the world average [2]. Migraine is characterized by recurrent headaches, varying duration from hours to days, often accompanied by a series of comorbidities, such as gastrointestinal symptoms, psychiatric disorders (i.e., depression and anxiety), and sleep disorders (i.e., insomnia, restless leg syndrome, sleep apnea, poor sleep quality and duration) [3, 4, 5, 6, 7]. These comorbidities, may last for a long period of time, contribute to the overall burden of migraine, cause disability, impair work, study and daily activities, and decrease quality of life. Therefore, migraine has been classified as a disabling disease [2, 8, 9].

Although the etiology, pathophysiology, and components involved with migraine is not well understood, multiple clinical studies have found that migraine patients often experience increased food allergy and sensitivity [10, 11]. Clinical studies have demonstrated improvements in migraine symptoms among patients with both allergies and migraine by using immunotherapy, allergic food restriction, and anti‐allergic therapies [12].

Food allergies can be categorized into three types: immunoglobulin E (IgE) mediated, non‐IgE mediated, or mixed [13]. There is research confirming that immunoglobulin G (IgG) can affect atopy, clinical symptoms, and the resolution of allergies beyond specific IgE [14]. IgG testing is based on the observation of high circulating serum concentrations of some IgG in certain atopic individuals. The high circulating serum concentrations may be due to certain subclasses of IgG or non‐IgE associated reactions that have been associated with in vitro degranulation of basophils and mast cells, the activation of complement cascade [15].

The diagnosis of a food allergy based on the specific IgG antibodies against food allergens is often performed when the classic methods of IgE mediated food allergy diagnostic cannot explain a patient's chief complaint, when symptoms were caused by the consumed foods [16].

IgG have been identified and reported to be associated with migraine attacks in susceptible individuals [17, 18] and IgG food sensitivity testing may be a beneficial tool for patients experiencing migraine headache symptoms [18, 19, 20]. Clinical studies have also found a noticeable upregulation in the immune system among the food specific IgG positive migraineurs is evidenced by increased inflammatory cytokines [19].

There is limited data on comorbidity symptoms and food specific IgG antibodies of migraine patients. We attempted to fill this research gap by collecting and analyzing real‐world data obtained from migraine patients in Harbin, Northeast China. Our study proposed to provide more evidence to reinforce the existing assumption regarding the correlation between migraine and its comorbidity symptoms using serum food specific IgG.

## 2 Materials and Methods

### 2.1 Patients

This cross‐sectional study involved migraine patients who visited the Department of Health Center of Screening and Prevention of Diseases, the First Affiliated Hospital of Harbin Medical University (Harbin, China) from October 28, 2020 to March 11, 2021. The inclusion criteria included: (1) female or male aged between 18 and 62; (2) with diagnosis of migraine more than 6 months and International Classification of Headache Disorders, 3rd edition (beta version) (ICHD‐3‐beta) was employed to diagnose migraine by two neurologists [21]; (3) ≥ 2 migraine attacks in the past 30 days. Subjects who had headache caused by other diagnosed diseases (i.e., hypertension, stroke, infectious disease, malignant/benign tumors, mental health disorders), peptic‐ulcer, autoimmune or current use of immunosuppressive drug or migraine preventive treatments were excluded.

The study was approved by the Ethics committee of the First Affiliated Hospital of Harbin Medical University (No. 201829). All participants provided written informed consent. Our research has been registered in Chinese Clinical Trial Registry (No. ChiCTR2000039278).

### 2.2 Clinical Assessment

The Clinical Assessment included: (1) age; (2) gender; (3) body height (cm); (4) body weight (kg); (5) with or without aura; (6) episodic migraine or chronic migraine; and (7) length of diagnosis. Additionally, specialized investigators assisted patients in filling out seven questionnaires to measure and evaluate the severity of the migraine and its comorbidities: (1) Migraine Disability Assessment (MIDAS) with headache days over the previous 3‐month period and Visual Analog Scale (VAS) (2) Headache Impact Test‐6 (HIT‐6) (3) Migraine‐Specific Quality of Life Questionnaire version 2.1 (MSQ) with raw dimension scores (4) Self‐Rating Anxiety Scale (SAS) (5) Self‐Rating Depression Scale (SDS) (6) Pittsburgh Sleep Quality Index (PSQI) (7) Gastrointestinal Symptom Rating Scale (GSRS).

### 2.3 Food‐Specific IgG Antibody Determination

We used enzyme‐linked immunosorbent assays (ELISA) to measure the serum concentration of 14 different types of food‐specific IgG (Bioeurope GmbH, Germany), according to the manufacturer's recommendations. A level of food‐specific IgG ≥ 50 U/mL was considered positive. Based on the results of the IgG concentration, participants who had at least one positive food were assigned into the IgG positive group, all others were assigned into the IgG negative group. All positive IgG concentrations were summed up as a total positive IgG for each patient.

### 2.4 Statistical Analysis

The sample size was estimated based on the feasibility considerations to meet the objective of this exploratory study. The demographic characteristics were summarized using descriptive statistics. The frequency of antigens for each IgG‐positive food and the corresponding IgG concentration were summarized using bar charts. Mean and standard deviation were used to describe continuous endpoints (i.e., time elapsed since diagnosis, MIDAS, etc.) and a two sample t test was used to test the significance in the difference between the groups of patients with positive and negative IgG foods. For the categorical endpoints (i.e., anxiety, depression, etc.), frequency and proportion were used and a Chi‐square test was performed to compare the difference between the two groups.

To assess the impact of frequency of IgG positive food on migraine and its comorbidities, simple linear regression models were built by using frequency of positive food as the factor. Regression coefficient and the 95% confidence interval (CI) were reported for the continuous endpoints, and odds ratio and 95% CI were reported for the categorical endpoints. Separate linear regression and logistic regression models were implemented to assess the association between total IgG concentration and each migraine endpoint. A significance level of 0.05 is used. All the statistical analyses were performed using Statistical Analysis Software (version 9.4; SAS Institute, Inc., Cary, NC, USA).

## 3 Results

### 3.1 Patient Characteristics

A total of 129 migraine patients who met the selection criteria were recruited. There were less males than females (43 vs. 86). The mean age was 40.1 (SD = 9.17). The length of the time since diagnosis ranged from 12 to 540 months (median length = 72 months). 7.8% of the patients had chronic headache and 10.9% had aura.

After blood tests, 98 (76.0%) patients who had at least one specific IgG antibody positive food (IgG ≥ 50 U/mL) were classified into the IgG positive group, the other 31(24.0%) patients were classified into the IgG negative group.

### 3.2 Characteristics of the IgG Positive Group

**Figure 1:**
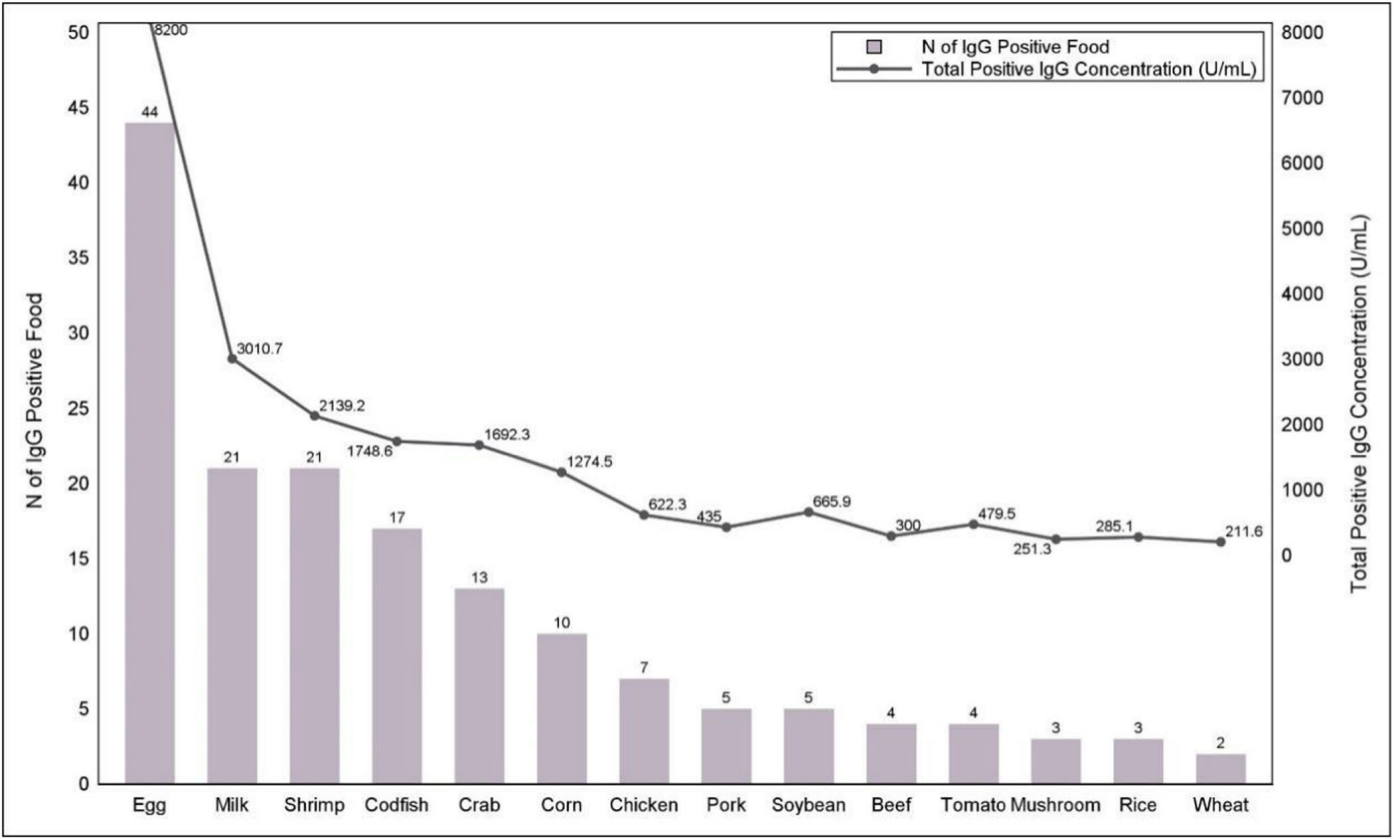
Frequency of IgG positive food and total positive IgG concentration for 98 participants with positive IgG.

For the participants in the positive IgG group, the total food specific IgG concentration ranged from 52.1 to 710.2 U/mL, with a mean of 217.5 U/mL (STD = 149.5 U/mL), and a median of 185.9 U/mL. In IgG positive group, the participants with one, two, and three or more than three positive food specific IgG were 49 (50.0%), 38 (38.8%), and 11 (11.2%), respectively. There was a large variation in the distribution of IgG food frequency and total IgG concentration (i.e., frequency ranges from 2 to 44, and IgG concentration ranges from slightly above 200 U/mL to over 8000 U/mL). Among the 98 participants, the top 7 prevalent food allergens were eggs (44.9%), cow's milk (21.4%), shrimp (21.4%), cold fish (17.3%), crab (13.3%), corn (10.2%), and chicken (7.1%). The first six foods listed above also had the higher total positive IgG concentration (The total IgG concentration for cow's milk is higher than that for shrimp). The last three foods with less IgG positive detection rate and lower total positive IgG concentration were all plant crops. Wheat had the least detection rate (2.0%) and lowest total IgG concentration as well (Figure 1). The mean concentration of IgG antibody against soybeans (133.2 U/mL) was just lower than cow's milk (143.4 U/mL) and eggs (186.4 U/mL).

### 3.3 Comparison of Characteristics Between the IgG Positive and Negative Groups

The IgG positive and negative groups were balanced in demographic characteristics, history of migraine, and more than half the migraine/comorbidity assessment endpoints. The scores of MIDAS, HIT‐6, MSQ, GSRS, and SAS were significantly higher for the patients in IgG positive group (46.8 vs. 34.5, 60.7 vs. 58.2, 31.0 vs. 24.8, respectively). Patients with positive IgG antibodies had a trend to have higher SDS score (43.3 vs. 39.3, *p*‐value: 0.0683) and experience depression (27.6% vs. 16.1%, *p*‐value: 0.1993) (Table 1).

**Table 1:** Comparison of the participants’ characteristics between food specific IgG positive and negative groups.

Characteristic	IgG positive (*N* = 98) [1]	IgG negative (*N* = 31) [1]	*p*‐value [2]
Age at enrollment	39.3 (8.4)	42.8 (11.0)	0.1100
Male *n* (%)	31 (31.6)	12 (38.8)	0.4663
BMI (kg/m^2^)	23.9 (3.7)	23.0 (3.5)	0.2249
aura, *n* (%)	12 (12.2)	2 (6.5)	0.3661
Chronic migraine, *n* (%)	9 (9.2)	1 (3.2)	0.2796
Time since diagnosis at enrollment (month)	100.3 (86.9)	73.4 (87.0)	0.1398
MIDAS	46.8 (31.7)	34.5 (14.8)	0.0037
N of days with migraine in past 3 months	18.9 (16.8)	14.7 (14.5)	0.1869
VAS	6.8 (1.3)	6.4 (1.5)	0.1595
HIT—6	60.7 (5.7)	58.2 (5.8)	0.0373
N of days with migraine in past 4 weeks	6.3 (5.5)	4.6 (4.5)	0.0919
MSQ	31.0 (12.3)	24.8 (9.9)	0.0061
SRS	30.4 (10.3)	26.7 (6.3)	0.0184
SDS	43.3 (10.7)	39.3 (10.3)	0.0683
Depression, *n* (%)	27 (27.6)	5 (16.1)	0.1993
SAS	42.5 (9.8)	38.4 (8.2)	0.0224
Anxiety, *n* (%)	22 (22.4)	4 (12.9)	0.2482
PSQI	7.8 (3.5)	7.4 (3.5)	0.5189
Poor sleeping, *n* (%)	44 (44.9)	14 (45.2)	0.9795

*Note:* [1] Continuous endpoints were summarized using mean (SD); categorical endpoints were summarized using count (%). [2] *p*‐value was from independent two group's *t* test for continuous endpoints and Chi square test for categorical endpoints.

### 3.4 The Association Between the Total Positive IgG Concentration, Number of IgG Positive Foods and Migraine and Its Comorbidities

The total IgG concentration showed positive relationships for most of the numeric endpoints, with higher concentration corresponding to worse conditions and greater disability of comorbidities. Statistical significance was observed in MIDAS, number of days with migraine in past 3 months, number of migraine days in past 4 weeks, MSQ, GSRS, SDS, and SAS, as shown in Table 2. Among those, MIDAS and number of migraine days in past 12 and 4 weeks presented highly statistical significance (i.e., *p* < 0.0001). An increase of 100 U/mL in total IgG concentration led to an average increase of 9.53 in MIDAS score (95% CI: 5.69–13.37, *p*‐value: < 0.0001). Among the four binary endpoints, the increase in total IgG concentration was related to the significant increase in the probability of experiencing depression. For every 100 unit increase in IgG concentration, the odds of experiencing depression increased 1.45 times (95% CI: 1.08, 1.95, *p*‐value: 0.0146). Total IgG concentration was significantly associated with chronic migraine, with higher IgG concentration suggesting higher probability of having chronic migraine (odds ratio: 2.03, 95% CI: 1.31, 3.15, *p*‐value: 0.0016).

**Table 2:** The association between the total positive IgG concentration, number of IgG positive foods, and migraine and its comorbidities.

	*N* of IgG positive food			Total IgG concentration	
	Category	Regression coefficient (95% CI)	*p*‐value	Regression coefficient (95%)	*p*‐value
MIDAS	2 vs. 1	7.30 (−5.97, 20.56)	0.2779	9.53 (5.69, 13.37)	< 0.0001
	≥ 3 vs. 1	27.17 (6.70, 47.65)	0.0098		
	≥ 3 vs. 2	19.88 (−1.13, 40.89)	0.0634		
N of days with migraine in past 3 months	2 vs. 1	1.55 (−5.27, 8.36)	0.6533	4.90 (2.86, 6.95)	< 0.0001
	≥ 3 vs. 1	19.02 (8.50, 29.54)	0.0005		
	≥ 3 vs. 2	17.47 (6.67, 28.27)	0.0018		
VAS	2 vs. 1	0.29 (−0.28, 0.86)	0.3151	0.08 (−0.18, 0.18)	0.9928
	≥ 3 vs. 1	0.55 (−0.33, 1.42)	0.2258		
	≥ 3 vs. 2	0.26 (−0.64, 1.16)	0.5656		
HIT—6	2 vs. 1	2.14 (−0.25, 4.52)	0.0791	0.71 (−0.04, 1.47)	0.0637
	≥ 3 vs. 1	3.89 (0.21, 7.58)	0.0387		
	≥ 3 vs. 2	1.76 (−2.03, 5.54)	0.3586		
N of days with migraine in past 4 weeks	2 vs. 1	0.17 (−2.05, 2.39)	0.8809	1.50 (0.82, 2.18)	< 0.0001
	≥ 3 vs. 1	6.33 (2.90, 9.76)	0.0004		
	≥ 3 vs. 2	6.16 (2.64, 9.68)	0.0008		
MSQ	2 vs. 1	4.90 (−0.23, 10.03)	0.0608	1.96 (0.33, 3.58)	0.0187
	≥ 3 vs. 1	10.71 (2.80, 18.62)	0.0085		
	≥ 3 vs. 2	5.81 (−2.31, 13.93)	0.1589		
GSRS	2 vs. 1	3.10 (−1.06, 7.26)	0.1426	2.04 (0.71, 3.38)	0.0031
	≥ 3 vs. 1	12.57 (6.14, 18.99)	0.0002		
	≥ 3 vs. 2	9.47 (2.88, 16.06)	0.0053		
SDS	2 vs. 1	2.92 (−1.49, 7.33)	0.1916	2.05 (0.67, 3.44)	0.0041
	≥ 3 vs. 1	10.55 (3.74, 17.35)	0.0027		
	≥ 3 vs. 2	7.62 (0.64, 14.61)	0.0327		
SAS	2 vs. 1	1.69 (−2.32, 5.71)	0.4039	1.37 (0.07, 2.67)	0.0386
	≥ 3 vs. 1	10.51 (4.31, 16.70)	0.0011		
	≥ 3 vs. 2	8.81 (2.46, 15.71)	0.0071		
PSQI	2 vs. 1	0.48 (−1.00, 1.96)	0.5207	0.08 (−0.39, 0.55)	0.7350
	≥ 3 vs. 1	2.25 (−0.04, 4.53)	0.0537		
	≥ 3 vs. 2	1.77 (−0.58, 4.11)	0.1377		

	Category	OR (95% CI)	*p*‐value	OR (95% CI)	*p*‐value
Depression	2 vs. 1	3.12 (1.10, 8.86)	0.0326	1.45 (1.08, 1.95)	0.0146
	≥ 3 vs. 1	10.50 (2.42, 45.49)	0.0017		
	≥ 3 vs. 2	3.37 (0.83, 13.64)	0.0892		
Anxiety	2 vs. 1	1.59 (0.55, 4.61)	0.3929	1.15 (0.85, 1.56)	0.3733
	≥ 3 vs. 1	4.27 (1.05, 17.46)	0.0433		
	≥ 3 vs. 2	2.69 (0.66, 10.92)	0.1676		
Poor sleep	2 vs. 1	1.20 (0.51, 2.81)	0.6748	0.92 (0.70, 1.20)	0.5386
	≥ 3 vs. 1	1.11 (0.30, 4.14)	0.8752		
	≥ 3 vs. 2	0.93 (0.24, 3.56)	0.9108		
Chronic Migraine	2 vs. 1	0.85 (0.14, 5.73)	0.8645	2.03 (1.31, 3.15)	0.0016
	≥ 3 vs. 1	8.76 (1.61, 47.73)	0.0121		
	≥ 3 vs. 2	10.29 (1.57, 67.45)	0.0151		

*Note:* N is the number of IgG positive food. Simple linear regression was used to estimate the regression coefficients for the categories of number of IgG positive foods (i.e., 1, 2, ≥ 3) and total IgG concentration. The estimates and 95% CIs were based on a 100 unit increase in total IgG concentration.

Meanwhile, significantly positive associations were found between the number of IgG positive foods and MIDAS, N of days in past 3 months, N of days in past 4 weeks, GSRS, SDS, SAS, and chronic migraine, when comparing the group with ≥ 3 foods to the groups of 1 and 2 foods. For each endpoint, the largest effect was observed among the group of ≥ 3 foods compared to the group of 1 food. For example, MIDAS score was 7.30 higher for the group with 2 IgG positive food (95% CI: −5.97, 20.56, *p*‐value: 0.2779), compared to the group with 1 IgG positive food, and this score was 19.88 higher for the group with ≥ 3 IgG positive food, compared to the group with 2 positive IgG food (95% CI: −1.13, 40.89, *p*‐value: 0.0634). Except for depression, most migraine and comorbidity endpoints were not significantly different when comparing the group of 2 foods to the group of 1 food.

## 4 Discussion

The results of this study found the difference in commodity symptoms between migraine subjects with and without positive food allergens, which further suggested that these comorbidity symptoms were not randomly occurring. Similar to migraine symptoms, IgG positive patients had significantly worse gastrointestinal and anxiety symptoms, as well as trend of higher rates of depression and SDS scores, compared with IgG negative migraine patients. More importantly, further analysis revealed that these variables were positively correlated with total positive IgG concentration and number of IgG positive foods (especially ≥ 3 vs. 1). Based on this, it is inferred that the food specific IgG antibody mediating hypersensitivity response may be one of the common pathological mechanisms of migraine and its gastrointestinal and psychiatric comorbidities (anxiety, depression). Specific foods cause delayed‐type hypersensitivity reactions and produce excess specific IgG antibodies. Allergens can activate trigeminal nerve inputs by enhancing the stimulation of the trigeminal nerve, causing degranulation of mast cells in the dura mater. This process may lead to local meningitis and cause intracranial pain. Vasodilatory substances and cytokines released by mast cells may play a role in the vasodilation phase of migraine and meningitis [20, 22]. In short, there might be a link between pro‐inflammatory potential of diet and migraine [9], and a series of immune reactions subsequently cause clinical manifestations of multiple systems.

Our study showed that the most common food allergies were eggs, cow's milk, and shrimp. This result is consistent with other epidemiologic studies of food allergy and sensitivity. In a study on Crohn's disease patients in Shanghai, the top five specific IgG positive rates were from egg, tomato, corn, rice, and cow's milk [23]. In Nanjing, a study tested 14 food specific IgG antibodies on 89 children with autism spectrum disorder, the results showed that the top six allergens with highest IgG concentrations were eggs, cow's milk, wheat, codfish, tomato, and soybean [16]. Slight variations observed in these results may be attributed to both genetic and environmental factors.

Some scholars have proposed that vegetables and fruits provide nutrients with anti‐inflammatory properties [24], while red meat and dairy products have inflammatory potential [25, 26, 27]. The findings in this study, which agree with this point of view, showed that foods with less IgG positive rates and lower total positive IgG concentrations were all plant crops, while the top five prevalent food allergens in this study were all of animal origin. Cow's milk and eggs with higher IgG antibody positive rates are the most frequently consumed daily foods by residents. Multiple studies have confirmed that eggs and dairy products have a strong stimulating effect on the immune system [23, 28, 29] and both are protein products that most people are exposed to in early childhood and consumed frequently, which lead to the accumulation of high‐level serum IgG antibody which activates the allergic reaction [30]. Antigens in shellfish, like crabs and shrimp, could initiate a high level of cross‐reactivity [31]. An interesting finding of this study is that the mean concentration of IgG antibody against soybeans was higher than that for all the other non‐protein allergens and was even higher than beef, even though soybean is a plant crop. This reinforced the preconceived notion that foods containing a high concentration of plant‐based protein also triggers an allergic reaction [24].

Foods have various components that may induce immune reactions, including the production of food‐specific immunoglobulin (Ig) [32]. IgG antibody‐mediated responses are associated with Type III hypersensitivity reactions, which are the body's regular reactions to food antigens. Once foods are ingested, the food antigens are usually digested and a tiny amount of allergens enter into the bloodstream. A certain concentration of IgG antibodies against food antigens was produced, maintained, and triggered the immune cells to release a variety of chemical factors that are involved in the allergic reaction process [33]. There are complexes of food antigens bound to specific IgGs circulating in the serum as well. These complexes are quickly cleared by the reticuloendothelial system, which explains the lower concentration of food specific IgG antibodies existing in healthy individuals and have almost no impact of pathogenicity. The excess of antibodies may lead to the immune complexes being deposited in blood vessels, they may also play a role in the pathogenesis of chronic intestinal inflammations by contributing to increased cuticular permeability [16, 17, 33, 34]. The discovery of bidirectional interaction between the gut and brain along the gut brain axis may help elucidate potential mechanisms. Gastrointestinal inflammation and neuroimmune regulation may have significant impacts on the pathological pathways of migraine [35, 36, 37].

Existing literature has revealed that dietary sensitivity based on IgG cause migraine [18]. Evidence from model studies found that the levels of IgG1 and IgG2 G0 were elevated in migraine patients with aura, indicating a relationship between migraine and IgG mediated immune response [38]. IgG antibody testing has been utilized in some clinical practices as a way to identify suspected food sensitivity and to develop diets targeted to reduce symptoms in migraine patients [25].

There is significant overlap between migraine and gastrointestinal symptoms. Nausea, vomiting, and delayed gastric emptying are part of migraine symptoms. Gastrointestinal disorders (GID), such as inflammatory bowel disease (IBD), abdominal disease (CD), and irritable bowel syndrome (IBS) [35, 36, 39] are commonly reported comorbidities in migraine patients [40]. In addition, the occurrence of abdominal migraines, defined as the simultaneous occurrence of migraines and abdominal symptoms during the migraine attacks, indicates that both affected systems share a common underlying mechanism [41]. There are a number of studies that have confirmed IgG hyperreactivity to food antigens in patients with GIDs [42, 43]. A cohort study on Bulgarian IBS patients found that food‐specific positive IgG antibodies were significantly higher in patients with IBS than in controls. Additionally, one‐third of IBS patients exhibited a low degree of chronic inflammation [43].

An online survey consisting of 2632 migraine patients in the United States showed that depression and anxiety were the most common comorbidities of migraine [3]. A study on adolescent patients with depression found that serum IgG and histamine levels of 14 different foods were significantly higher than those for the healthy control group. In addition, over 80% of patients exhibit long‐term food intolerance which leads to high permeability of the blood–brain barrier, this in turn, may play a part in the pathogenesis of Major depressive disorder [34]. In our study, the increase in total IgG concentration was related to the significant increase in the probability of experiencing anxiety, depression and chronic migraine, confirming the positive correlation between them.

Our results did not support the hypothesis that IgG positive foods were associated with sleep disorders. One of the possible reasons is that the factors that affect sleep are more diverse and complex, and the pathogenesis behind the relationship between the two may also involve some overlapping pathophysiology and common anatomical structures in the brain [44]. The relationship between migraine and sleep is bidirectional: on the one hand, sleep disorders (such as excessive sleep, insufficient sleep, or irregular sleep) are known triggers and risk factors for migraine; and on the other hand, migraine can interfere with the patient's sleep quality [7]. It is worth noting that although statistical significance was not achieved, the ranges of 95% CI of three odds ratios comparing different allergens, all include estimates that are clinically meaningful (i.e., 0.51–2.81, 0.30–4.14, and 0.24–3.56, respectively). Therefore, further clinical study that examines the association and etiology regarding the common pathogenesis between them is warranted.

In the diagnosis and treatment of migraine patients, the high prevalence of related comorbidities such as depression and anxiety, as well as sleep disorders and gastrointestinal dysfunction, is often overlooked. However, in reality, comorbidities can increase migraine related disabilities and reduce quality of life [5]. Similarly, the interaction between food sensitivity and medication treatment should be taken seriously [45]. For migraine patients, especially those with poor standard treatment outcomes, doctors should perform a more detailed evaluation of migraine comorbidities and fully investigate the possible common mechanisms of these multisystem diseases, such as IgG mediated food allergies discovered in this study.

There are several limitations to this study that cannot be overlooked. First, this study was carried out during the COVID‐19 epidemic in China. Although there was no confirmed cases of COVID‐19 infection among the 129 subjects during the research period, their lives were greatly affected. China has implemented mandatory measures such as community control and closure of some public places to prevent further escalation of the epidemic [46]. During the period of home medical observation, people did not engage in outdoor activities, their physical activity decreased, and their work and social habits changed. Such sudden and significant public health crisis might have exacerbated anxiety, depression, and sleep disorders in the subjects of this study [47, 48]. The prevalence of anxiety and sleep disorders in both food specific IgG positive and negative migraine patients in this study was not only higher than that in Zhao et al.'s 2014 study of 1113 patients with episodic migraine in China (the prevalence of anxiety and sleep disorders was 6.8% and 7.9%, respectively) [9] but also higher than that in Shi et al.'s study of 56,932 general population subjects in China in 2020 (the prevalence of anxiety, depression and insomnia was 31.6%, 27.9%, and 29.2%, respectively) [46]. Despite the influences caused by the COVID‐19 epidemic, our study still found noteworthy positive results indicating that delayed‐type hypersensitivity caused by food specific IgG positive foods may be an independent risk factor for migraine and its gastrointestinal and psychiatric comorbidities. Second, using questionnaires to collect information may lead to bias as it relies on participants’ recall ability and educational level. Third, this study used a cross‐sectional design which limited the inference of the etiology of migraine and its comorbidities that may be caused by IgG mediated food allergy. Therefore, future work is required to understand the causal sequence of relationships in a longitudinal study with larger sample size.

## 5 Conclusion

This study found that compared with food specific IgG negative migraine patients, positive patients had significantly worsened headache, gastrointestinal and anxiety symptoms. The effect of food specific IgG antibodies on the severity of migraine and its comorbidities are antibody‐quantity and IgG‐concentration dependent. The current findings might suggest a possible role of food specific IgG antibodies in the pathogenesis of migraine. However, more research is needed in this field, such as the inflammatory mechanisms involved, changes in neurotransmitters, and intestinal permeability and microbiota.

## Author Contributions

Zhi‐Ming Zhao collected the data, performed experiments, wrote original draft, acquired funding. Mei‐Mei Yang performed statistical analyses and investigation. Xian‐Shu Zhao collected and curated the data and investigation. Fu‐Jun Wan collected and curated the data. Bao‐Li Ning performed the execution and collected the data. Li‐Ming Zhang reviewed and edited draft. Jun Fu designed the experiments, supervised, and acquired funding. All authors read and approved the final manuscript.

## Ethics Statement

The ethics and protocol of this research study were assessed and approved by the Ethics committee of the First Affiliated Hospital of Harbin Medical University (No. 201829).

## Conflicts of Interest

The authors declare no conflicts of interest.

## Data Availability

Data can be requested from the corresponding author by E‐mail after publication.
